# Deep learning approaches for non-coding genetic variant effect prediction: current progress and future prospects

**DOI:** 10.1093/bib/bbae446

**Published:** 2024-09-13

**Authors:** Xiaoyu Wang, Fuyi Li, Yiwen Zhang, Seiya Imoto, Hsin-Hui Shen, Shanshan Li, Yuming Guo, Jian Yang, Jiangning Song

**Affiliations:** Monash Biomedicine Discovery Institute and Department of Biochemistry and Molecular Biology, Monash University, Melbourne, VIC 3800, Australia; Monash Data Futures Institute, Monash University, Melbourne, VIC 3800, Australia; South Australian immunoGENomics Cancer Institute (SAiGENCI), Faculty of Health and Medical Sciences, The University of Adelaide, Adelaide, SA 5005, Australia; School of Public Health and Preventive Medicine, Monash University, Melbourne, VIC 3004, Australia; Genome Center, Institute of Medical Science, The University of Tokyo, Minato-ku, Tokyo 108-8639, Japan; Collaborative Research Institute for Innovative Microbiology, The University of Tokyo, Bunkyo-ku, Tokyo 113-8657, Japan; Department of Materials Science and Engineering, Faculty of Engineering, Monash University, Clayton, VIC 3800, Australia; School of Public Health and Preventive Medicine, Monash University, Melbourne, VIC 3004, Australia; School of Public Health and Preventive Medicine, Monash University, Melbourne, VIC 3004, Australia; School of Life Sciences, Westlake University, Hangzhou, Zhejiang 310030, China; Westlake Laboratory of Life Sciences and Biomedicine, Hangzhou, Zhejiang 310024, China; Monash Biomedicine Discovery Institute and Department of Biochemistry and Molecular Biology, Monash University, Melbourne, VIC 3800, Australia; Monash Data Futures Institute, Monash University, Melbourne, VIC 3800, Australia

**Keywords:** non-coding variants, variant effect prediction, machine learning, deep learning

## Abstract

Recent advancements in high-throughput sequencing technologies have significantly enhanced our ability to unravel the intricacies of gene regulatory processes. A critical challenge in this endeavor is the identification of variant effects, a key factor in comprehending the mechanisms underlying gene regulation. Non-coding variants, constituting over 90% of all variants, have garnered increasing attention in recent years. The exploration of gene variant impacts and regulatory mechanisms has spurred the development of various deep learning approaches, providing new insights into the global regulatory landscape through the analysis of extensive genetic data. Here, we provide a comprehensive overview of the development of the non-coding variants models based on bulk and single-cell sequencing data and their model-based interpretation and downstream tasks. This review delineates the popular sequencing technologies for epigenetic profiling and deep learning approaches for discerning the effects of non-coding variants. Additionally, we summarize the limitations of current approaches in variant effect prediction research and outline opportunities for improvement. We anticipate that our study will offer a practical and useful guide for the bioinformatic community to further advance the unraveling of genetic variant effects.

## Introduction

Most heritable diseases, such as Alzheimer’s disease, psychiatric disorders, coronary heart disease, and Parkinson’s disease, stem from the intricate interplay of genetic and environmental factors [1]. Genetic factors play a pivotal role in influencing individual susceptibility to these diseases. Emerging studies underscore the significance of variant effects of various diseases, such as Frazer *et al*. (Evolutionary model of variant effect [EVE]) [2], MacArthur *et al.* [3], and Lappalainen *et al.* [4]. Leveraging genome-wide association studies (GWAS), previous investigations have revealed the existence of over 200,000 genetic variants, linked to more than 3000 human traits [5]. Deciphering the function impact of these variants and their contributions to phenotypic changes is crucial for advancing precision medicine.

In the past decades, there has been a growing appreciation of the significance of non-coding sequences that were once considered ‘junk’ DNAs due to their unknown functions [6]. In the human genome, more than 98% is non-coding [7], harboring the vast majority (>90%) of disease-associated single nucleotide polymorphisms (SNPs) [8]. In contrast to protein-coding variants that directly impact protein structure or function, non-coding variants exert their influence through the modulation of gene regulatory processes. Therefore, understanding the function of non-coding variants can help unravel the gene regulatory process and decode human diseases.

In this review, we explore state-of-the-art deep learning methods for identifying the effects of the non-coding variants and emphasizing their application in downstream tasks and model interpretation. Additionally, we critically point out the limitations of current methodologies, followed by proposing promising directions for future research.

## Non-coding region, gene regulation, and epigenetic modification

**Figure 1:**
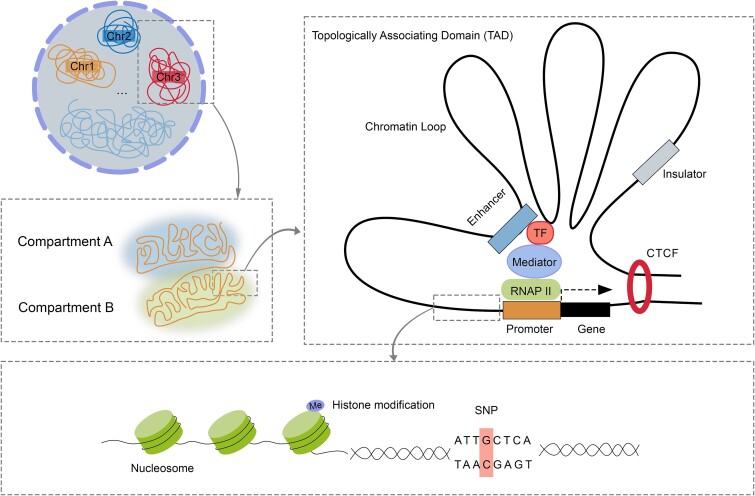
An overview of hierarchical organization of chromatin structure and the gene regulatory process.

Non-coding regions refer to the DNAs that do not encode proteins, comprising a large proportion of the genomes. Non-coding regions often contain regulatory elements that regulate gene transcription, such as enhancers and promoters. The gene regulatory process operates across multiple levels, encompassing transcriptional, post-transcriptional, translational, and post-translational mechanisms. Non-coding variants primarily impact gene regulatory process through regulatory elements and chromatin status at the transcriptional level. These regulatory elements can be broadly classified into proximal/cis-regulatory elements (e.g., the promoter, enhancer, insulator, and silencers) and distal/trans-regulatory elements (e.g., Transcription Factor [TF] and non-coding RNA) that located at a considerable distance from the transcription start site (TSS) [9]. Understanding the functional significance of regulatory elements and unravelling their contributions to disease pathogenesis is a complex task. This involves extensive epigenetic modifications, encompassing histone modification, transcription factor (TF) binding, DNA accessibility, and genome topology (Fig. 1). Therefore, previous computational methods considered multiple epigenetic modifications (mentioned earlier) to comprehensively quantify non-coding variant effects.

Histones are a class of proteins rich in lysine and arginine residues, forming nucleosomes by involving DNA wrapped around them, constituting the basic structural units of chromatin. Histone modifications, such as acetylation of arginine or lysine residues and lysine methylation, play a crucial role in gene regulation and expression. Histone acetylation, one of the most common histone modifications, regulates gene expression by reshaping the structure of chromatin, and some studies suggest that acetylation may be associated with gene activation and local chromatin opening [10]. Histone methylation, another critical modification, encompasses diverse forms with primary modification sites located predominantly on histone H3. Histone modification has been revealed to be closely linked to early embryonic development [11]. The NIH Roadmap Epigenomics Consortium proposed five core types of histone modifications [12], including H3K4me1 or H3K27ac (enhancer), H3K4me3 (promoter), H3K36me3 (transcribed regions), H3K27me3 (Polycomb repression), and H3K9me3 (heterochromatin).

Transcription factors are proteins that play a crucial role in transcription and the regulation of gene expression by binding to TF binding sites in cis-regulatory regions like enhancers and promoters. These TFs can influence cell differentiation and mutations within the TF binding sites that have been linked to multiple human diseases, including heart disease [13], diabetes [14], and chronic kidney disease [15].

Moreover, chromatin accessibility serves as a crucial indicator for evaluating the chromosomal state at a genome-wide scale. It represents the extent to which chromatinized DNA is available for interaction with other regulatory elements, providing insights into over 90% of TF binding sites [16] and potential regulatory gene locations. Typically, chromosomes exhibit intricate folding, exposing DNA only during the transcription process and facilitating the binding of other regulatory elements.

Furthermore, 3D chromatin architecture is intricately linked to gene regulation, influencing gene expression across various scales, encompassing nucleosome-nucleosome interaction, chromatin loops, topologically associating domains (TADs), and compartments [17]. Regulatory elements can physically interact with distant elements through chromatin loops, despite the considerable element distance. For instance, the promoter–enhancer loop. A recently noteworthy revelation, known as TADs, is the partitioning of chromatin into sub-megabase-scale domains based on the frequency of chromatin interactions, within which enhancer-promoter interactions predominantly occur. Additionally, numerous DNA-binding proteins, including CCCTC-binding factor (CTCF), yin yang 1 (YY1), and zinc finger protein 143 (ZNF143), in conjunction with the mediator complex, play essential roles in shaping chromatin structure and governing gene regulation.

## Experimental technologies for interpretation of non-coding variants

The advancement of next-generation sequencing technologies, coupled with the decreasing costs of sequencing techniques, has resulted in a substantial increase in the accumulation of genome and epigenetic data. This presents a remarkable opportunity to transform precision medicine, disease diagnosis, and evolutionary biology, by unraveling the latent potential of non-coding DNA. The continuous advancements in sequencing technology have greatly enriched our understanding of genomics. The effects of variants can be observed in plenty of aspects, including TF binding, histone modification, chromatin accessibility, and chromatin structure. Among these, gene expression stands out as the most intuitionistic measurement for assessing variant effects. In this section, we provide an overview of the fundamental sequencing technologies employed for genome and epigenetic profiling in Table 1.

**Table 1:** Sequencing technologies for investigating gene regulation and variant effects

Data types	Methods	Pros	Cons
Gene expression	DNA microarray [18]	Relatively low cost, no length bias	Rely on the known sequences
	RNA-seq [19]	Higher resolution, lower limit of detection	Relatively high cost, length inherent bias
	CAGE [20]	Can identify the TSS activity, high reproducibility	Only for total mature RNA, detection bias for long-lived transcripts
DNA accessibility	DNase-seq [21]	Can examine regulatory regions	Limited by the intrinsic bias for DNase I
	ATAC-seq [22]	High efficiency, low cellular input requirement	Tn5 transposase binding bias, relatively high cost
	MNase-seq [23]	Maximize the preservation of the original chromatin structure	Easily lose some weak binding sites
	FAIRE-seq [24]	Not rely on enzymes	Lower resolution and signal-to-noise ratio
Histone modification and TF binding	Chip-seq [25]	Higher resolution, less noise, and greater coverage	Require a large number of cells, antibody requirement, potential for epitope masking
	CUT&RUN [26]	Lower background noise, low cellular input requirement	Require end polishing and adapter ligation
	CUT&Tag [27]	Lower background noise, low cellular input requirement, low cost	Not suitable for low abundance targets, TF, and chromatin-associated proteins
	DAP-seq [28]	No sample-specific reagents requirement, retain tissue/cell-line specific secondary modification	Relatively higher failure rate, not suitable for low abundance targets, only for TF binding events
3D genome	Hi-C [29]	Detects all intra- and inter-chromosomal interactions	Lower resolution
	Micro-C [30]	Higher resolution	Fewer long-range or inter-chromosomal interactions

The most widely employed sequencing technologies for identifying transcript abundances encompass DNA microarray [18], RNA-seq [19], and Cap Analysis Gene Expression (CAGE) [20]. DNA microarray technology quantifies the abundance of known genes or RNA species through binding targets to specific oligonucleotide probes. In contrast, RNA-seq utilizes high-throughput next-generation sequencing technologies not only to quantify RNA abundance but also to discover novel sequences and identify alternative splicing events. CAGE is a crucial method for measuring transcript abundances and comprehensively identifying the TSS at the single-nucleotide resolution. In comparison to DNA microarray and RNA-seq, CAGE not only measures gene expression level but also defines the promoter landscape. CAGE measures RNA expression by capturing the 5’end of capped RNA, corresponding to the TSS. This method provides valuable insights into the transcription dynamics, thereby facilitating variant effect prediction.

Chromatin immunoprecipitation sequencing (ChIP-seq) [25] is a powerful approach for researchers studying the interaction between DNA and DNA-binding proteins, such as TFs and histone with specific modifications, in the pan-genomic regulatory landscape. ChIP-seq has also been widely adopted for TF binding and histone modification identification. Antibodies participated in the ChIP-seq process by binding to the protein of interest present in the DNA sequences. This specific binding enriches the target DNA sequence, which is then processed into a DNA library. Therefore, the accuracy of ChIP-seq highly depends on the quality and specificity of antibodies. The genome segments are sequenced using high-throughput sequencing technologies and the sequenced fragments are aligned to the reference genome to identify the location of binding protein. Moreover, Cleavage under targets and release using nuclease (CUT&RUN) [26] and Cleavage under targets and tagmentation (CUT&Tag) [27] are also powerful methods for the target protein, with a high signal-to-noise ratio. To address the limitations associated with specific reagent requirements, such as antibodies or primers, DNA Affinity Purification Sequencing (DAP-seq) [28] has been recently developed. It enables the identification of genome-wide TF binding events while preserving tissue- or cell line-specific chemical modifications. However, DAP-seq has a relatively low success rate and is not well-suited for quantifying low-abundance proteins.

In addition to the TF binding and histone modification, chromatin accessibility is also crucial for studying gene regulation. Principal methods for measuring chromatin accessibility include DNase I hypersensitive site sequencing (DNase-seq) [21], Assay for transposase-accessible chromatin with high-throughput sequencing (ATAC-seq) [22], Micrococcal nuclease digestion with high-throughput sequencing (MNase-seq) [23], and Formaldehyde-Assisted Isolation of Regulatory Elements sequencing (FAIRE-seq) [24]. To examine the chromatin state, most sequencing technologies aim to identify unprotected DNA sequences in the open chromatin region. Several enzymes are adopted in different approaches, such as DNA endonuclease DNase I (DNase-seq), micrococcal nuclease (MNase-seq), and hyperactive Tn5 transposase (ATAC-seq). Unlike other sequencing methods, FAIRE-seq targets accessible DNA regions based on their lack of histone cross-linking. Coupled with high-throughput sequencing technology, these approaches can effectively identify DNA accessibility.

Advancements in sequencing technology over the past decade have significantly improved our understanding of chromosome conformation and nuclear architecture. A major breakthrough was the establishment of chromosome conformation capture (3C) technologies, enabling the investigation of chromosome conformation [31]. Subsequently, various 3C derivatives were introduced, including the widely used highest-throughput 3C (Hi-C) [29] and the micrococcal nuclease chromosome conformation assay (Micro-C) [30]. Hi-C is the most extensively employed method for comprehending genome-wide chromatin folding and interactions. Micro-c utilizes micrococcal nuclease (MNase) instead of restriction enzymes, allowing for the capture of interactions at a finer resolution due to MNase’s ability to generate more uniform fragmentation. However, Micro-C may lose some long-range inter-chromosomal interactions.

### Single-cell sequencing technologies

Advancements in single-cell isolation and barcoding technologies have propelled significant development in single-cell sequencing technologies in recent years. In contrast to bulk sequencing technologies, single-cell sequencing is able to capture diverse cell states and developmental trajectories. Single-cell sequencing technology excels in profiling multi-omics data, encompassing gene expression, DNA modification, histone modification, DNA accessibility, and 3D genome architecture as summarized in Table 2.

**Table 2:** Single-cell sequencing technologies

	Sequencing technologies
Gene expression	scRNA-seq, Smart-seq, Quartz-seq, Drop-Seq, DroNc-seq, CEL-seq, C1-CAGE, RamDa-seq
DNA modification	scBS-seq, scRRBS
Histone modification	scCHIP-seq
DNA accessibility	scNOME-seq, scATAC-seq, scDNase-seq
3D genome	scHi-C

To assess gene expression (transcript expression), various single-cell sequencing methods based on whole-transcriptome amplification (WTA) have emerged. These methods include Smart-seq, Quartz-seq, CEL-seq, and C1-CAGE. Additionally, RamDa-seq is designed for measuring non-poly(A) transcripts, including long non-coding RNAs and enhancer RNAs. Complementing the WTA methods, several microdroplet-based protocols such as Drop-Seq and DroNc-seq, along with microwell-based methods like Seq-Well, have been further proposed.

Beyond gene expression, single-cell sequencing technologies are versatile for epigenetic profiling from multiple aspects, including DNA modification (single-cell bisulfite sequencing [scBS-seq] [32], single-cell reduced-representation bisulfite sequencing [scRRBS] [33]), DNA accessibility (single-cell nucleosome occupancy and methylome sequencing [scNOME-seq] [34], Single-cell sequencing assay for transposase-accessible chromatin [scATAC-seq] [35], single-cell DNase sequencing [scDNase-seq] [36]), Histone modification (single-cell chromatin immunoprecipitation DNA sequencing [scChIP-seq] [37]) and 3D genome (single-cell high-resolution 3C methodologies [scHi-C] [38]).

Moreover, driven by advancements in high-throughput technologies, there has been an exponential increase in high-quality sequencing data and publicly accessible databases, facilitating the comprehensive assessment of variant effects on a larger scale. These databases are summarized in Table 3.

**Table 3:** Publicly accessible epigenetic profile databases

Database	Histone modification		DNA accessibility		Gene expression		3D genome		Species	URL	Reference
	B	S	B	S	B	S	B	S			
ENCODE	✓		✓	✓	✓	✓	✓		Human/Mouse	https://www.encodeproject.org/	[39]
Roadmap	✓		✓						Human	https://www.ncbi.nlm.nih.gov/geo/roadmap/epigenomics/	[12]
IHEC	✓				✓				Human	https://epigenomesportal.ca/ihec/index.html	[40]
Cistrome DB	✓		✓						Human/Mouse	http://cistrome.org/db/	[41]
FANTOM5					✓		✓		Human/Mouse	https://fantom.gsc.riken.jp/5/	[42]
4DN	✓	✓	✓	✓	✓		✓	✓	Multiple	https://data.4dnucleome.org/	[43]
GEO	✓	✓	✓	✓	✓	✓	✓	✓	Multiple	https://www.ncbi.nlm.nih.gov/geo/	[44]
Single Cell Portal				✓		✓			Multiple	https://singlecell.broadinstitute.org/single_cell	[45]
PanglaoDB						✓			Human/Mouse	https://panglaodb.se/index.html	[46]
Single cell expression atlas						✓			Multiple	https://www.ebi.ac.uk/gxa/sc/home	[47]
HCA data portal						✓			Human/Mouse	https://data.humancellatlas.org/	[48]

Abbreviations: Roadmap: Roadmap epigenomics database. IHEC: International Human Epigenome Consortium. 4DN: 4D Nucleome Data Portal. HCA: Human cell atlas. B: Bulk sequencing technologies. S: Single-cell sequencing technologies.

## Deep learning approaches for analyzing the effects of non-coding variants

Given the significance of non-coding regions, numerous studies have dedicated substantial efforts to unravel the effects of non-coding variants [49]. Experimental methods for identifying variant effects are low-throughput, time-consuming, and labor-intensive. In this regard, deep learning approaches prove highly effective in deciphering variant effects, considering the remarkable complexity of gene expression and transcriptional regulation. The impact of non-coding variants is often described as alterations in the regulatory process and corresponding gene expression. A comprehensive and robust model can offer novel insights into the mechanisms of variant effects and their associations with human diseases and traits. In recent years, an increasing number of studies have leveraged cutting-edge deep learning-based technologies for predicting variant effects, driven by the emergence of high-throughput sequencing, public epigenetic data, and artificial intelligence advancements [50–53]. We provide an overview of the current deep learning approaches for analyzing the effects of non-coding variants in Table 4. To further evaluate the usability of non-coding variant models, we tested the models’ running time to generate the prediction results based on the web server or NVIDIA GeForce RTX 3090 (Table S1). The sequence length and model complexity are considered as the primary factors that influence the processing time.

**Table 4:** Summary of deep learning methods for non-coding variants

Name	Year	Type	TF	Histone mark	DNA accessibility	CAGE	3D genome	DNA methylation	Input length	Genome	Algorithms	Species	Cell-type	Website
DeepBind	2015	Peak	509	–	–	–	–	–	14-101 nt	GRCh37/hg19	CNN	human, mouse	multiple	http://tools.genes.toronto.edu/deepbind/ (not available)
Deepsea	2015	Peak	690	104	125	–	–	–	1000 bp	GRCh37/hg19	CNN	human	multiple	http://deepsea.princeton.edu/job/analysis/create/
Basset	2016	Peak	–	–	164	–	–	–	500-1000	GRCh37/hg19	CNN	human	164	NA
DanQ	2016	Peak	690	104	125	–	–	–	1000 bp	GRCh37/hg19	CNN, BLSTMs	human	multiple	NA
CpGenie	2017	Peak	–	–	–	–	–	50	500 bp	GRCh37/hg19	CNN	human	GM12878	NA
Basenji	2018	Genetic track	–	2307	949	973	–	–	131 kb	GRCh37/hg19	CNN, dilated CNN	human	multiple	NA
DeFine	2018	Peak	148	–	–	–	–	–	300 bp	GRCh37/hg19	CNN	human	K562, GM12878	http://define.cbi.pku.edu.cn/
DeepFIGV	2019	Peak	–	3 histones for 75 individuals	69	–	–	–	300-2000 bp	GRCh37/hg19	CNN	human	multiple	NA
NCNet	2019	Peak	690	104	125	–	–	–	1000 bp	GRCh37/hg19	Bottleneck Residual then Recurrent Network Model	human	multiple	NA
ExPecto	2019	Peak	690	978	334	–	–	–	2000 bp	GRCh37/hg19	CNN + spatial transformation module + Linear model for tissue-specific expression	human	multiple (218)	http://hb.flatironinstitute.org/expecto
TBiNet	2020	Peak	690	–	–	–	–	–	1000 bp	GRCh37/hg19	CNN, Attention BiLSTM	human	multiple	http://tbinet.korea.ac.kr/ (Not available)
Basenji2	2020	Genetic track	2131	1860	684/228	638/357	–	–	131 kb	GRCh38/hg38	CNN, dilated CNN	human mouse	multiple	NA
DeepFun	2021	Peak	4795	1536	1548	–	–	–	1000 bp	GRCh37/hg19	CNN (similar to Basset)	human	225	https://bioinfo.uth.edu/deepfun/
Enformer	2021	Genetic track	2131	1860	684	638	–	–	196 kb	GRCh38/hg38	CNN, residual block, transformer	human mouse	multiple	NA
Sei	2022	Peak	9471	10,064	2373	–	–	–	4000 bp	GRCh38/hg38	Residual dual linear and nonlinear paths; residual dilated convolutional network; spatial basis function	human	multiple	https://hb.flatironinstitute.org/sei
GraphReg	2022	Genetic track	–	H3K4me3, H3K27ac	DNase-seq	–	Hi-C, HiChIP, Micro-C, HiCAR	–	6 M	GRCh37/hg19	CNN, dilated CNN, Graph attention network	human mouse	multiple	NA

^a^Year: Year of Publication.

**Figure 2:**
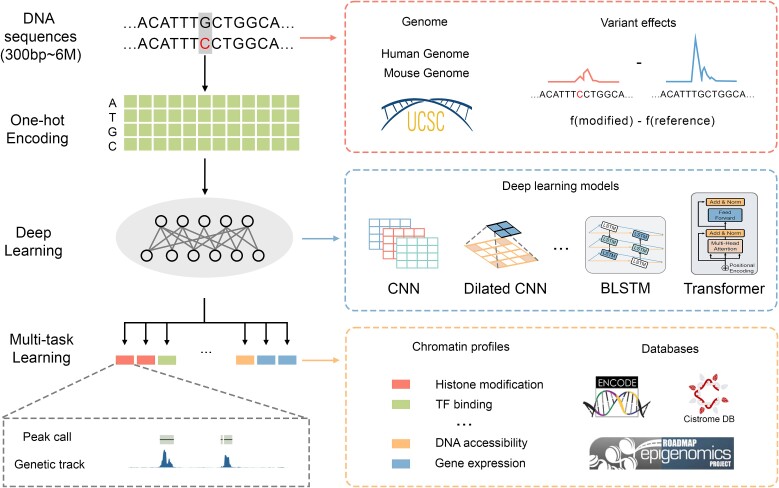
A typical workflow illustrating the application of machine learning approaches in predicting variant effects.

**Figure 3:**
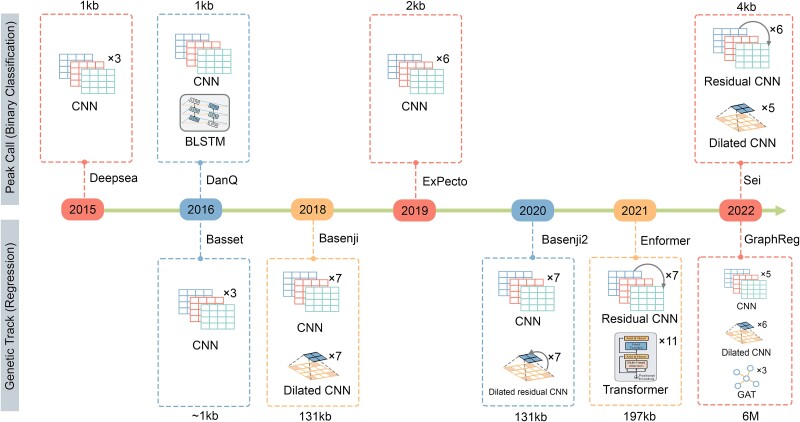
Deep learning methods for non-coding variant effects.

In this review, we focus on the sequence-based model, particularly those with DNA sequences as the sole input. Figure 2 illustrates the typical workflow for predicting non-coding variant effects and Fig. 3 showcases the evolution of the deep learning methods in this domain. To predict non-coding variant effects, the input data of these models, namely DNA sequences, typically range from 300 bp (base pairs) to 6 Mb (megabase pairs), targeting descriptors such as histone modifications, TF binding, DNA accessibility, and gene expression. Commonly employed architectures for these models include convolutional neural networks (CNNs), dilated CNNs, bidirectional long short-term memory (BLSTM), and Transformers.

CNNs are a type of deep neural network widely used in computer vision and sequence analysis [54]. A fundamental element of CNNs is the convolutional kernel, which slides over the input data to capture underlying patterns. Specifically, in DNA sequence analysis, this kernel can detect motifs within the DNA sequence. CNN layers are typically used as the initial layers to extract features from DNA sequences. Dilated CNNs, a variant of standard CNNs, incorporate dilated kernels that have spaces between kernel elements. Such design allows them to capture distant features, thereby enabling them to capture long-distance dependencies within the data. These networks are typically used following CNN layers to integrate the extracted information. Apart from convolutional layers, long short-term memory (LSTM) networks are another popular machine learning algorithm for processing DNA sequences [55, 56]. LSTMs have a set of gates that manage the flow of information across long sequences. The forget gate discards irrelevant information from the previous time step while allowing relevant information to persist within the network. Given the bidirectional nature of DNA, which consists of two polynucleotide chains, bidirectional LSTMs, incorporating both forward and backward LSTMs, are more commonly used than unidirectional LSTMs due to their excellent ability to effectively handle this two-way data flow.

Transformer is another type of deep learning method that is also widely used to model sequence data [57]. It includes the multi-head attention mechanism that uses multiple self-attention layers to learn the relationships between the tokens (i.e., learned motifs). The attention formula can be expressed as follows:


$$ Attention\left(Q,K,V\right)= softmax\left(\frac{Q{K}^T}{\sqrt{d_k}}\right)V $$

where the $Q$, $K$, V stand for query, key, and value, respectively, all of which are generated by the same feature vector. ${d}_k$is the dimensionality of the key. To integrate the positional information of the sequence, Transformers use the positional encoding to weight each token.

Additionally, a multi-task learning strategy was employed to integrate the information from different epigenetic prediction tasks. In contrast to single-task learning, multi-task learning typically shares some common layers and parameters within a single model, thereby facilitating the simultaneous training of multiple tasks. Each task utilizes specific parameters to address the differences between tasks. In the context of epigenetic profiling, the shared parameters might include the common motifs learned across different tasks and potential relationships between these motifs in DNA sequences. Accordingly, this approach can enable the overall model to more comprehensively learn the gene regulatory process from multiple tasks.

For the non-coding variants model, we categorize deep learning methods into peak-based and genetic track-based methods based on the target formats.

### Peak-based binary classification approaches

Peak-based methods center around identifying the presence or absence of peaks within designated regions. These methods leverage peak calling algorithms to define target peaks, ultimately addressing a binary classification problem. In 2015, Alipanahi *et al*. introduced, DeepBind, a deep learning framework using the position weight matrices (PWMs) of sequences for identifying sequence specificities of DNA- and RNA-binding proteins. Shortly after, Zhou *et al*. proposed DeepSEA [53], the first fully sequence-based deep learning model for non-coding variant effect prediction. Unlike previous approaches, DeepSEA takes the DNA sequence as input, instead of the known motifs or other handcrafted features. DeepSEA employs a deep convolutional network (CNN) with three convolution layers and one fully connected layer. DeepSEA is trained on the whole genome functional annotation on the single-nucleotide resolution with the input of 1000 bp DNA sequence. Multi-tasking learning is adopted for large-scale chromatin-profiling data, which involves the 690 TF binding profiles, 104 histone-mark profiles, and 125 DNase I hypersensitive site profiles, to share the parameters. The variant effect was measured by a log2 fold change of odds. Subsequently, to help understand gene accessibility and interpret the function of non-coding regions, Kelley *et al*. introduced Basset [58] to predict DNA accessibility in 164 cell types Using a CNN model with three convolutional layers and two fully connected layers. Basset is able to capture the DNA binding motifs by PWMs in their initial convolution layer. The emergence of new deep learning networks also facilitated the identification of non-coding variant effects. DanQ [59] achieved a better performance than DeepSEA by combining the CNN model and BLSTM. Specifically, in addition to CNN layers, DanQ utilized one convolutional layer to capture the motif and a BLSTM layer to learn the grammar of regulation. In 2019, Zhou *et al*. proposed ExPecto [50] (DeepSEA2.0), an expansion of the DeepSEA model, which contains three key components: a deep learning model for epigenetic profile prediction, a spatial transformation module, and tissue-specific expression models. Compared with DeepSEA, ExPecto increased training epigenetic profiles from 919 to 2002, incorporating 334 DNase-seq profiles, 978 histone modification profiles, and 690 TF binding profiles for 218 tissues and cell types. The input length expanded to 2000 bp and thus enhanced information capture substantially. The deep learning framework employed in ExPecto resembles DeepSEA, with two additional modules included for spatial and tissue-specific information. TBiNet [60] focuses on the TF binding sites prediction and leveraging a combination of CNN, attention mechanism, and BiLSTM. DeFine [61] is a CNN model designed for the TF binding intensity prediction using TFs Chip-seq data from K562 and GM12878 cell lines. DeepFIGV [62], sharing the framework of Basset, uses distinct training data, including three kinds of histone modification profiles and one DNA accessibility profile. Inspired by deep residual networks, NCNet [63] enhances identification performance by incorporating bottleneck residual and recurrent neural networks based on DanQ. Moreover, DeepFun [64] offers a user-friendly web server, employing a CNN model akin to the Basset [58] with three CNN layers and two fully connected layers.

A recent breakthrough is Sei proposed by Chen *et al*. [51], a deep-learning framework designed for predicting regulatory activity and elucidating the mechanisms underlying disease-related variants. Sei emerges as the most comprehensive model for the regulatory network to date. It leverages an extensive dataset encompassing 9471 TF binding profiles, 10 064 histone mark profiles, and 2373 chromatin accessibility profiles, encompassing a total of 21 907 cis-regulatory elements. The identification of sequence classes from the whole genome contributes to a broader understanding of global regulatory activities. Sei has successfully unveiled potential mechanisms for cell type-specific regulatory activities and the identification of disease-associated variants based on the sequence class.

### Genetic tracks-based regression approaches

This section delves into regression approaches that utilize quantitative genomic profiles (genomic tracks) for model training, contrasting with binary targets (peak calls) employed in previous studies. The use of genomic tracks offers a richer source of information compared to peak calling. To expand the Basset model [58], Kelley *et al*. extended their research to encompass a broader array of epigenetic and expression profiles, including TF binding, chromatin accessibility, and histone modification. The training dataset encompasses 949 DNase-seq profiles, 2307 histone modification profiles, and 973 CAGE-seq profiles. They predicted genomic profiles at a finer resolution through introducing a deep learning model named Basenji [65]. Basenji incorporates four convolutional layers and seven dilated convolutional layers that capture up to 131 kb distal influences. Consequently, the test accuracy of this model improved with an increased receptive field width.

While earlier studies predominantly focused on human genomic information, incorporating data from multiple species in model training can enhance the transfer of annotations from other species to the human genome. Recognizing the potential of cross-species information, Kelley introduced Basenji2 [66], a model trained simultaneously on human and mouse data. Basenji2 utilized 638 CAGE profiles, 684 DNase-seq and ATAC-seq profiles, and 3991 Chip-seq profiles in human and 357 CAGE profiles. 228 DNase-seq and ATAC-seq profiles and 1058 Chip-seq profiles for mouse. The initial stage of Basenji2 employs a CNN model to extract motifs from the DNA sequence. Subsequently, residual networks are incorporated, featuring a dilated convolutional layer to capture information in long sequences and mitigate the vanishing gradient issue. Finally, a linear transform is involved to predict human and mouse regulatory activities.

Harnessing the potency of Transformer, Avsec *et al*. proposed Enformer, one of the most powerful deep learning algorithms renowed for its excellence in natural language processing, [52]. This transformer-based model utilized the same training dataset as Basenji2, marking a significant stride in DNA sequence analysis akin to language processing. Following a pattern similar to prior research, the initial module of Enformer comprises seven convolutional layers, complemented by an architecture featuring eleven transformer layers. A noteworthy enhancement in Enformer is the expanded receptive field, allowing for the integration of long-range information. In its final stage, Enformer incorporates organism-specific heads for human and mouse data, enabling adaptability across different species. In a related vein, Zrimec *et al*. used deep learning methods to unravel gene expression [67]. Karbalayghareh *et al.* introduced a novel non-coding variant effect prediction method, GraphReg, that breaks new ground in the field. This groundbreaking approach integrates 3D genome information, amalgamating CNN, dilated CNN, and graph attention network components to predict epigenetic profiles and gene expression.

In the realm of regulatory activity prediction, CNN stands out as the predominant architecture, particularly as the first layer. The initial convolution layer, equipped with various position-weight matrices, excels in motif detection. Empirical evidence suggests that convolutional layers combined with max-pooling are highly effective for extracting TFs motifs. An essential consideration in non-coding variants prediction is the choice between binary (peak call) and quantitative (genetic tracks) targets. One of the foremost challenges is managing long DNA sequence and their corresponding deep neural networks. To address this, two main strategies are adopted: refining the model structure and lowering the resolution of the target. This evolution has seen the transition from CNN to dilated CNN and then to the transformer, thereby enlarging the receptive field of the input in prediction model from 1 kb to 196 kb. Some models, like Enformer and Basenji2, have reduced resolution to better accommodate distal regulatory elements. Furthermore, multi-tasking learning is frequently employed in non-coding variant prediction to leverage shared parameters across different tasks, enhancing overall model performance.

### 3D genome structure prediction

In addition to epigenetic modification, the regulatory activities of the genome are notably influenced by its 3D structure. Certain mutations can alter chromatin contacts, thereby affecting the regulation of gene expression. To predict genome folding, recent deep learning methods like DeepC [68], Akita [69], and Orca [70] have been developed. Specifically, DeepC [68] utilizes transfer learning for chromatin folding prediction. It features a convolutional module with max pooling, followed by a dilated convolutional module, culminating in a fully connected layer that synthesizes the learned information for chromatin folding prediction. Fudenbery and Kelley, along with their colleagues, proposed Akita [69], which also predicts 3D genome folding. Inspired by Basenji, the first module of Akita, comprises 1D convolutional and dilated residual convolutional layers. To mimic chromatin interactions, the 1D output of this model is then converted into 2D format by averaging the outputs across genomic bin pairs. Subsequently, multiple symmetric dilated residual 2D convolutional layers are employed. Finally, the model compares its predicted map with the target map. Both Akita and DeepC process ~1 Mb DNA sequence as input but differ in their data processing strategies; DeepC encodes Hi-C data as a vector, whereas Akita converts 1D input into a 2D map. Another important contribution to this field is Orca [70], proposed by Zhou in 2022. Orca is a multi-scale deep learning model with a multi-resolution encoder and cascading decoder, designed to predict the chromatin interaction frequencies across scales from kilobase to whole chromosome.

### Cis-regulatory element prediction

The identification of non-coding variant effects can also be approached through the prediction of cis-regulatory elements. We summarize the deep learning methods for the cis-regulatory elements in Table 5. Currently, most of the methods are sequence-based models that primarily concentrate on the identification of a single type of cis-regulatory element. For the promoter prediction, previous methods only take the DNA sequence with length normally less than 600 bp as the input, such as DeeProPre [75] and HMPI [70]. For the Enhancer identification and strength prediction, a state-of-the-art method is ADH-Enhancer [79, 81], which is an attention-based hybrid deep learning model that combines the CNN, LSTM and attention module. Compared with the promoter and enhancer, the identification of the silencer is more complex. However, the mechanism of the silencer is unclear. Therefore, various methods are proposed to incorporate more genetic information to facilitate the prediction. For instance, DeepICHS [84] improves the accuracy of prediction through incorporating epigenetic profiling data. DeepICHIS uniquely integrates DNA sequence information with multi-omics data, including histone modification, TF binding, and DNA accessibility, to identify cell-specific silencers. Another example is SilenceREIN [85], which is the first attempt to combine the DNA sequence and chromatin structure information for silencer identification. SilenceREIN leverages the structure information of the regulatory element interaction network to locate the silencer on anchors of chromatin loops. In summary, deep learning methods have been widely used for single cis-regulatory element identification. However, the impact of structure information and the epigenetic profile for cis-regulatory element identification has been overlooked.

**Table 5:** Summary of deep learning methods for cis-regulatory elements identification

Regulatory elements	Methods	Year	Algorithm	Input length	Inputs	Reference
Promoter	CNNProm	2017	CNN	81 bp	DNA sequences	[71]
	DeeReCT-PromID	2019	CNN	600 bp	DNA sequences	[72]
	DeePromoter	2019	CNN, BiLSTM	300 bp	DNA sequences	[73]
	HM-Prom	2021	CNN	300 bp	DNA sequences	[74]
	HMPI	2022	CNN	251 bp	DNA sequence, DNA structure	[75]
	DeeProPre	2022	CNN, BiLSTM, Attention	300 bp	DNA sequence	[75]
Enhancer	DeepEnhancer	2016	CNN	300 bp	DNA sequences	[76]
	BiRen	2017	CNN, GRU, BRNN	1000 bp	DNA sequences	[77]
	iEnhancer-GAN	2021	CNN, GAN	200 bp	DNA sequences	[78]
	SENIES	2022	CNN	200 bp	DNA sequences, DNA structure	[79]
	iEnhancer-BERT	2022	CNN, Bert, Transfer learning	200 bp	DNA sequence	[80]
	ADH-Enhancer	2024	CNN, LSTM, Attention	200 bp	DNA sequence	[81]
Silencer	Huang et al.	2019	SVM	–	DNA sequences, Histone modification, DNA accessibility	[82]
	DeepSilencer	2021	CNN	–	DNA sequences	[83]
	DeepICSH	2023	CNN, Attention, Autoencoder	200 bp	DNA sequence, Histone modification, DNA accessibility, TF binding	[84]
	SilenceREIN	2024	CNN, GraphSAGE, MLP	600 bp	DNA sequence, Histone modification, TF binding,	[85]

^a^Year: Year of Publication.

### Phenotypic variant effect prediction

In this review, we primarily focus on computational methods that predict the effect of non-coding variants at the transcription level. However, given the advancements in deep learning models for predicting the effects of phenotypic variants, this section also explores models that can be used to predict phenotypic variant effects. Phenotypic variants are more complex than non-coding counterparts, involving multiple processes including post-transcriptional, translational, and post-translational processes. There are increasing research efforts devoted to predicting phenotype-associated variant effects, incorporating diverse types of information such as individual variants, structural information, and coding sequences. For example, DeepPerVar [86] is a CNN-LSTM multi-modal model that integrates individual variants with epigenetic data to improve phenotype-specific predictions. GenNet is another deep learning approach that utilizes individual variants to predict phenotypes [87]. Sequence UNET leverages the protein sequences to predict the variant frequency, with the purpose of better understanding their impacts on phenotypes [88]. Additionally, another method Cue integrates structural information to predict multiple types of complex structural variants that have been linked to genetic diseases [89].

### Predicting with the single-cell sequencing technologies

In the realm of single-cell sequencing, which provides a granular view of cellular states and development, developing effective deep-learning models is challenging due to data’s scarcity and relatively limited volume. Recent advancements have brought forward several deep-learning approaches for deciphering gene regulation at the single-cell level, and we outlined these approaches in Table 6. Distinct from methods for bulk variant analysis, these models often employ a crucial strategy: leveraging variant effect models trained on bulk sequencing data to refine single-cell prediction. For instance, Huatuo [90] employs the Expecto model to extract chromatin embeddings and uses an XGBoost model for predicting single-cell transcriptome profiles, particularly focusing on scRNA-seq based gene expression. Similarly, Seq2cell leverages the pre-trained Enformer for epigenetic embedding generation, enabling the integration of distal information up to 196 kb in its analysis.

**Table 6:** Summary of deep learning methods based on single-cell sequencing technologies

Name	Year	Targets	Algorithms	Inputs length	Species	Cell-type
ScFAN	2020	TF binding	CNN	1000 bp	Human	3
scBasset	2022	DNA accessibility	CNN	1344	Human/mouse	multiple
nvwa	2022	Gene expression	CNN, RNN	13 kb	8	multiple
scEpoLock	2022	DNA accessibility	CNN	1000 bp	Human	5
Huatuo [90]	2023	Gene expression	Transfer learning, XGBoost	40 kb	Human	multiple
Seq2cell [91]	2023	Gene expression	Transfer learning	196 kb	Human	multiple
scXpresso	2023	Gene expression	CNN	10.5 kb	Human	multiple

^a^Year: Year of Publication.

## Interpretation and Down-stream tasks

The breakthrough advances in deep learning models for non-coding variants have transformed the comprehension of gene regulation and disease-associated variants. Due to the qualitative nature of variant effects, the interpretation and evaluation of non-coding variants are conducted through various tasks. These include motif identification, prioritization of functional SNPs, cell-specific gene expression analysis, identification of regulatory elements, and analysis of disease-associated loci, as detailed in Table 7. Drawing from existing research, we classify these tasks into two broad categories: model-based interpretation and downstream tasks.

**Table 7:** Interpretation and down-stream tasks of non-coding variants models

Name	Year	Motif analysis	In silico saturation mutagenesis	Functional SNP prioritization	Regulatory elements analysis	Disease-associated loci
DeepBind	2015	✓				
Deepsea	2015		✓	✓		
Basset	2016		✓	✓		
DanQ	2016	✓	✓	✓		
CpGenie	2017	✓		✓		
Basenji	2018		✓	✓	✓	✓
DeFine	2018	✓		✓		
DeepFIGV	2019	✓		✓		
NCNet	2019					
ExPecto	2019		✓			
TBiNet	2020	✓				
Basenji2	2020			✓	✓	
DeepFun	2021	✓	✓			
Enformer	2021		✓	✓	✓	
Sei	2022					✓
GraphReg	2022	✓			✓	✓

^a^Year: Year of Publication.

## Model-based interpretation

### Motif analysis

**Figure 4:**
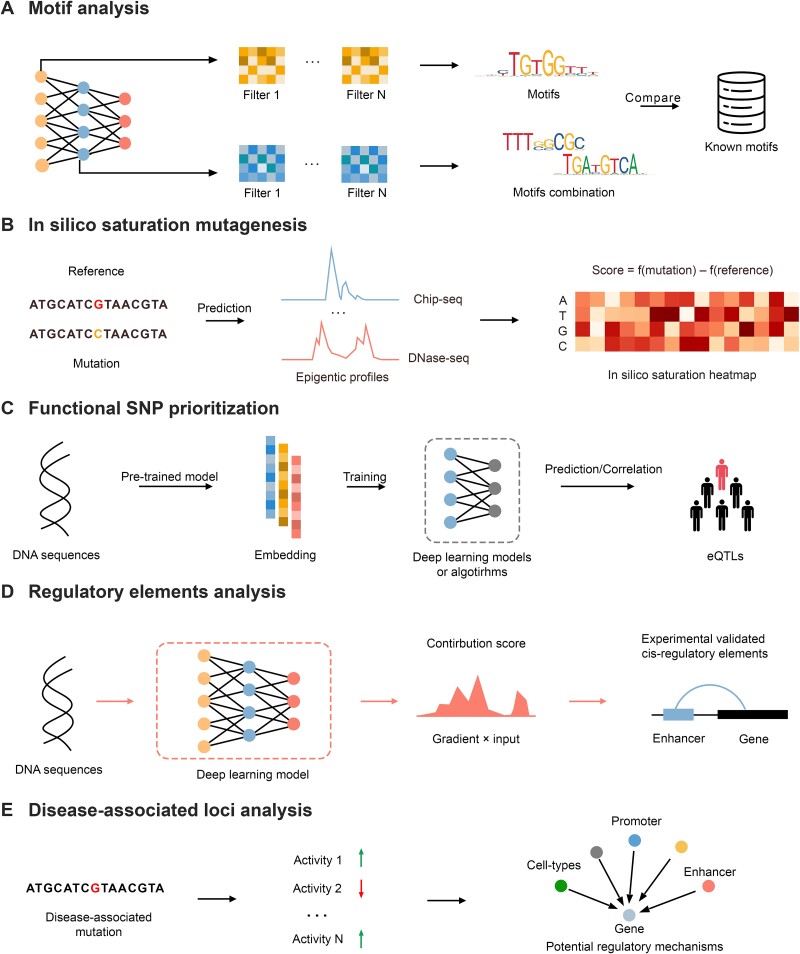
Interpretation and down-stream tasks.

Motifs-based methods are a predominant strategy for uncovering hidden patterns in sequence-to-activity models, particularly in CNN models. As illustrated in Fig. 4A, the first layer neurons of the convolutional network are adept at detecting motifs by screening the DNA sequence with a PWM. Some studies have extracted these motifs and compared them with TF binding motifs from databases like JASPAR [92]—an open-source repository containing thousands of non-redundant TF binding profiles. These investigations confirm that motifs identified by the model align with experimentally validated motifs. In layers beyond the first convolutional layer, such as the second layer or deeper, the neurons can represent interactions between motifs or longer motif structures.

### In silico saturation mutagenesis

Another model-based interpretation method is implemented based on the in-silico saturation mutagenesis, which is among the most crucial approaches for variant effect models. Given the challenges of directly quantifying the variant effect, the prevailing strategy simulates variant effects by involving substantial alterations in multiple epigenetic profiles resulted from a base substitution. The effects of the variant can be defined in various formats. For instance, in DeepSEA, the impact of a based substitution is quantified by the log2 fold change:


$$ effect={\log}_2\left(\frac{P_0}{1-{P}_0}\right)-{\log}_2\left(\frac{P_1}{1-{P}_1}\right) $$

where ${P}_0$ and ${P}_1$ are the probability of the original sequence and the predicted mutated sequence, respectively.

## Down-stream tasks

### Functional SNP prioritization

Genome-wide association studies (GWAS) serve as a cornerstone for discovering disease genes. GWAS have revealed thousands of genetic variants associated with human diseases and traits [93]. Traditional GWAS analyses assume linear relationships between genetic variants and phenotypes. However, the causal effects between variants and phenotypes are often complex and nonlinear. Additionally, some variants are in linkage disequilibrium, displaying strong correlations with other variants. This correlation can pose challenges in pinpointing disease-causing mutations. Artificial intelligence methods can enhance the discovery of disease susceptibility loci and the understanding of variant effects. Computational models can identify Expression quantitative trait loci (eQTL) [94] without measuring thousands of individual gene expression profiles. Functional SNP prioritization employs two strategies: (i) Computing the correlation between predicted variants and eQTL variant effects using methods such as Spearman correlation and the Signed Linkage Disequilibrium Profile regression score. (ii) Constructing a model that utilizes embeddings from the non-coding variant prediction model to identify eQTLs.

### Gene regulation elements identification

Deep learning methods not only aid in predicting non-coding variant effects but also provide a global interpretation of the gene regulatory network. By analyzing predicted changes in each profile, potential regulatory mechanisms and their corresponding cis-regulatory elements can be inferred. This offers new perspectives on regulatory element identification, including enhancer-promoter interaction identification, cell type or tissue-specific enhancers identification, and distal regulatory element identification. Enhancer plays a crucial role in mammalian transcriptional control, influencing cell differences and the timing of the cell cycle. Several studies have demonstrated the ability of deep learning models on prioritizing enhancers with high accuracy [52], leveraging the attention mechanism or the gradient × input as contribution score. Apart from enhancers, insulator elements, usually located near TAD boundaries, are also essential for gene regulation.

### Disease-associated loci analysis

Unraveling the mechanisms underlying diseases and pinpointing disease-associated genetic variants is a formidable challenge. Leveraging deep learning models trained on large-scale datasets, several studies have begun to shed light on disease mechanisms from new angles. Analyzing changes in rich chromatin profiles utilized by non-coding variant models can provide insights into the disease-associated pathways impacted by these variants. For example, Basset [58] defined SNP Accessibility Difference profiles to pinpoint causal variants associated with autoimmune diseases. Similarly, Basenji [65] demonstrated how certain loci associated with autoimmune diseases and blood cell traits could alter transcription or chromatin profiles of genes. Additionally, Sei offered a comprehensive map of mutation effects, classifying and quantifying the direction and magnitude of variant effects at the sequence class level, encompassing all 853 regulatory diseases.

## Conclusions and future perspectives

In this review, we have centered on exploring sequence-to-activity models concerning the impact of non-coding variants at the transcriptional level. The past few years have witnessed a surge in efforts to unravel the complexities of gene regulation and the effects of genetic variants. In the face of the intricate nature of gene regulation and the rapidly expanding trove of genomic data, deep learning has risen as a pivotal tool. Its exceptional proficiency in detecting complex patterns has facilitated several groundbreaking discoveries, ranging from gene expression prediction to the identification of crucial regulatory elements. Despite these advancements, there are still opportunities within this field remains for further enhancement and development.

The evolution of deep learning methods in genomics is characterized by increasing reliance on longer DNA sequence inputs, cross-species training, and expansive training datasets. Analysis of recent models reveals that longer DNA sequences enable more comprehensive information capture. This is crucial because gene expression can be influenced by trans-regulatory elements located distantly from the TSS. However, challenges still lie in effectively managing and interpreting long-range information as input lengths increase. Therefore, it is essential to develop robust deep learning models capable of handling these long-range interactions with minimal information loss. Another promising area of development is the integration of 3D genome data, specifically chromatin interaction information. While traditional studies have focused primarily on sequence distances, incorporating the spatial gene positioning of genes—critical for understanding gene regulation—could significantly enhance model accuracy and insights.

Furthermore, current models for predicting the effects of individual genomic variants on gene expression may not always achieve the desired level of accuracy [95]. For instance, Sasse *et al*. [95] observed the discrepancies in gene expression prediction accuracy when applying Enformer to genomic data from 839 individuals, particularly in the predicted direction (i.e., increase or decrease) of gene expression changes. Some of the predictions displayed a negative correlation with observed gene expression, suggesting that the model performed poorly for predicting the expression of certain genes. Furthermore, the input sequence length used by some models exceeded 131 kb, leading to a considerably large potential sequence space. To improve accuracy in gene expression analysis, it is vital to incorporate individual variant information. This approach deviates from traditional methods that rely on a single reference genome, emphasizing the need to consider locus-specific variants that can influence gene expression. In addition to DNA-based sequence analysis, post-transcriptional processes, including RNA-protein and RNA–RNA interactions, also play a significant role in impacting the effects of variants. However, to our knowledge, there are currently no methods available that take into consideration the effect of such post-transcriptional processes. Therefore, a comprehensive approach that accounts for the RNA abundance in combination with DNA analysis is crucial and desirable to better model and interpret the impact of non-coding variants on gene expression processes.

**Key Points**

- We reviewed the deep learning methods for the effect of non-coding variant prediction, including the methods based on bulk sequencing and single-cell sequencing. Additionally, we comprehensively introduced the sequencing methods and publicly available databases relevant to the non-coding variants prediction.
- Current sequence-to-activity methods for non-coding variants focus on the incorporation of long-range information and large-scale epigenetic information across different cell types or species. The model-based interpretation and downstream tasks can facilitate the understanding of the variant effects.
- Accurately predicting the effect of non-coding variants remains challenging. Future research that integrates more information, such as long-range interaction information, individual variants, and RNA interaction information, is crucial for fully understanding the gene regulatory process and variants effects.

## Supplementary Material

Supplementary_document_bbae446
