# Supplementary material for: Deep learning approaches for non-coding genetic variant effect prediction: current progress and future prospects
Source: Brief Bioinform. 2024 Sep 13;25(5):bbae446. doi: 10.1093/bib/bbae446 (PMC11401448; doi:10.1093/bib/bbae446)
Supplement: Supplementary_document_bbae446 [file supplementary_document_bbae446.docx]

**Deep learning approaches for non-coding genetic variant effect prediction: current progress and future prospects**

Xiaoyu Wang^1,2^, Fuyi Li^3^, Yiwen Zhang^4^, Seiya Imoto^5,6^, Hsin-Hui Shen^7^, Shanshan Li^4^, Yuming Guo^4^, Jian Yang^8,9,*^, Jiangning Song^1,2,*^

^1^Monash Biomedicine Discovery Institute, Monash University, Melbourne, VIC 3800, Australia;

^2^Monash Data Futures Institute, Monash University, Melbourne, VIC 3800, Australia;

^3^South Australian immunoGENomics Cancer Institute (SAiGENCI), Faculty of Health and Medical Sciences, The University of Adelaide, Adelaide, SA 5005, Australia;

^4^School of Public Health and Preventive Medicine, Monash University, Melbourne, VIC 3004, Australia;

^5^Genome Center, Institute of Medical Science, The University of Tokyo, Minato-ku, Tokyo 108-8639, Japan;

^6^Collaborative Research Institute for Innovative Microbiology, The University of Tokyo, Bunkyo-ku, Tokyo 113-8657, Japan;

^7^Department of Materials Science and Engineering, Faculty of Engineering, Monash University, Clayton, VIC 3800, Australia;

^8^School of Life Sciences, Westlake University, Hangzhou, Zhejiang 310030, China;

^9^Westlake Laboratory of Life Sciences and Biomedicine, Hangzhou, Zhejiang 310024, China.

^*^To whom correspondence should be addressed:

Jiangning Song, Biomedicine Discovery Institute and Department of Biochemistry and Molecular Biology, Monash University, Victoria 3800, Australia. Email: [Jiangning.Song@monash.edu](mailto:Jiangning.Song@monash.edu).

Jian Yang, School of Life Sciences, Westlake University, Hangzhou, China. Email: [jian.yang@westlake.edu.cn](mailto:jian.yang@westlake.edu.cn).

**Keywords:** non-coding variants; machine learning; deep learning

**Running Head**: non-coding variant effect prediction

**Table S1.** Running time of the non-coding variant models.

| Name | Testing time | Platform | Code/Website |
| --- | --- | --- | --- |
| DeepBind | - | - | <http://tools.genes.toronto.edu/deepbind/>  (Not available) |
| Deepsea | ~1m per 1000 variants | Web server | http://deepsea.princeton.edu/job/analysis/create/ |
| Basset | <1s per 1000 variants | NVIDIA GeForce RTX 3090 | https://github.com/davek44/Basset |
| DanQ | <1s per 1000 variants | NVIDIA GeForce RTX 3090 | https://github.com/uci-cbcl/DanQ |
| Basenji | ~1m per 1000 variants | NVIDIA GeForce RTX 3090 | <https://www.github.com/calico/basenji> |
| DeFine | - | - | <http://define.cbi.pku.edu.cn/> (Not available) |
| ExPecto | ~1m per 1000 variants | Web server | http://hb.flatironinstitute.org/expecto |
| Basenji2 | ~1m per 1000 variants | NVIDIA GeForce RTX 3090 | https://github.com/calico/basenji |
| DeepFun | ~5s per variants | Web server | <https://bioinfo.uth.edu/deepfun/> |
| Enformer | ~27s per variants | T4 GPU(Colab) | https://github.com/google-deepmind/deepmind-research/tree/master/enformer |
| Sei | ~1m per 1000 variants | Web server | <https://hb.flatironinstitute.org/sei> |
| GraphReg | ~8s | NVIDIA GeForce RTX 3090 | https://github.com/karbalayghareh/GraphReg/ |

*The performance was tested on the platforms provided by the author or NVIDIA GeForce RTX 3090.

**Table S2.** List of acronyms used in this review.

| Category | Acronym | Definition |
| --- | --- | --- |
| Molecular biology | LD | Linkage Disequilibrium |
|  | eQTL | expression Quantitative Trait Loci |
|  | SNP | Single Nucleotide Polymorphism |
|  | Me | Methylation |
|  | RNAP II | RNA polymerase II |
|  | TAD | Topologically Associating Domain |
|  | TF | Transcription Factor |
|  | GWAS | Genome-Wide Association Studies |
|  | SAD | SNP Accessibility Difference |
|  | TSS | Transcription Start Site |
|  | CAGE | Cap Analysis of Gene Expression |
|  | Hi-C | Highest-throughput Chromosome conformation capture |
|  | 3C | Chromosome Conformation Capture |
|  | Micro-C | Micrococcal nuclease Chromosome conformation assay |
|  | WTA | Whole-Transcriptome Amplification |
|  | ChIP | Chromatin immunoprecipitation |
| Deep learning | CNN | Convolutional Neural Network |
|  | RNN | Recurrent Neural Network |
|  | BLSTM | Bidirectional Long Short-Term Memory |
|  | LSTM | Long Short-Term Memory |
|  | PWM | Position-Weight Matrix |
